# Associations between school-based fluoride mouth-rinse program, medical-dental expense subsidy policy, and children's oral health in Japan: an ecological study

**DOI:** 10.1186/s12889-024-18156-y

**Published:** 2024-03-12

**Authors:** Takafumi Yamamoto, Sakura Kiuchi, Miho Ishimaru, Hideki Fukuda, Tetsuji Yokoyama

**Affiliations:** 1 Department of Health Promotion, National Institute of Public Health, Saitama, Japan; 2 Department of International and Community Oral Health, Graduate School of Dentistry, Tohoku University, Miyagi, Japan; 3 Frontier Research Institute for Interdisciplinary Sciences, Tohoku University, Miyagi, Japan; 4 The Institute of Education, Tokyo Medical and Dental University, Tokyo, Japan; 5 National Institute of Public Health, Saitama, Japan

**Keywords:** Claim data, Floride mouth rinsing program, Medical policy, Universal approach

## Abstract

**Background:**

Dental caries are a common non-communicable disease among children. As a public health measure at the prefectural level, school-based fluoride mouth-rinse (S-FMR) program, medical/dental expense subsidy policies, and other factors may reduce the incidence of dental caries and tooth loss. Prefectures focusing on promoting oral health policies may promote both, but the interaction effect of implementing both subsidy policies and S-FMR at the prefectural level on caries prevention has not yet been examined.

**Methods:**

We conducted an ecological study using two-wave panel data, prefecture-level aggregated data in Japan for 2016 and 2018. Coefficient and 95% confidence intervals (CI) were calculated for the dependent variables for oral health using mixed-effects linear regression analysis adjusted for possible confounders. Two dependent variables were used; the standardized claim ratio (SCR) of deciduous tooth extraction and 12-year-olds’ decayed, missing, or filled permanent teeth (DMFT). Four independent variables were S-FMR, the SCR of dental sealants, prefectural income per person, and subsidy policy in three models: co-payment until children enter elementary school (*n* = 23), no co-payment until children enter elementary school (*n* = 7), and co-payment continuing beyond elementary school (*n* = 17). The effects of six interaction terms, each representing a unique pairing from the four independent variables, were individually calculated.

**Results:**

S-FMR was negatively associated with the SCR of deciduous tooth extractions and DMFT (coefficient = -0.11, 95% CI -0.20; -0.01 and coefficient = -0.003, 95% CI -0.005; -0.001, respectively). No co-payment until children enter elementary school was positively associated with the SCR of deciduous tooth extraction compared to co-payment until children enter elementary school(coefficient = 11.42, 95% CI 3.29; 19.55). SCR of dental sealants was positively associated with the SCR of deciduous tooth extractions (coefficient = 0.12, 95% CI 0.06; 0.19) but negatively associated with DMFT (coefficient = -0.001, 95% CI -0.003; -0.0001). Per capita prefectural income was positively associated with the SCR of deciduous tooth extractions(coefficient = 0.01, 95% CI 0.001; 0.02). No interaction was found between S-FMR and the subsidy policy at both outcomes.

**Conclusion:**

High S-FMR utilization and no co-payment until children enter elementary school were associated with fewer deciduous tooth extractions. Also, S-FMR and dental sealant were associated with decreased DMFT.

**Supplementary Information:**

The online version contains supplementary material available at 10.1186/s12889-024-18156-y.

## Introduction

Dental caries is a common non-communicable disease affecting adults and children [1, 2], and previous studies suggest its prevalence was associated with socioeconomic status (SES) [3, 4]. Protecting children's oral health from economic inequaliies is a crucial issue in dental public health. One approach is the use of fluoride, such as tap water fluoridation or pit and fissure sealant application, which has been shown to be effective in preventing childhood caries [5, 6]. Tap water fluoridation is recognized as one of the top 10 public health achievements of the twentieth century by the US Centers for Disease Control and Prevention [7]. In Japan, several municipalities intervene in children's oral health through school-based fluoride mouth-rinse program (S-FMR). Previous studies have suggested that S-FMR reduces dental caries among children [8–10].

Another measure to protect children's oral health is the introduction of universal health coverage into the dental care system to eliminate inequalities in dental care access [11, 12]. Universal health coverage reduces out-of-pocket costs for medical and dental care, thereby encouraging community-dwellers to use them [13]. In Japan, dental care is provided under the universal health insurance system [14]. Additionally, each prefecture has its own policies to assist with medical and dental expenses for children [15]. These policies involve contributions from the prefecture or municipality to reduce out-of-pocket costs for residents. Through this subsidy policy, the co-payment for children is kept lower than that for adults (30%). However, the amount of assistance and eligibility requirements vary by region (the self-payment rate was 0% to under 30% across prefectures). Given that the price elasticity of dental care costs is higher than that of medical care costs [16], residents in areas where the co-payment rate is kept low may increase their dental visits compared to those in other areas.

While both S-FMR and medical and dental subsidy policies may be effective in protecting children's oral health, prefectures that focus on oral health policies may promote both, and there may be an interaction between them. This study aimed to: 1) examine the effects of S-FMR, the subsidy policy, the prevalence of dental sealants, and regional-level income on children's oral health, 2) determine whether there are interaction effects among community-level factors influencing children's oral health, specifically between S-FMR, subsidy policies, the prevalence of dental sealants, and regional economic conditions.

## Methods

### Study setting

This ecological study targeted all 47 prefectures in Japan, using data from multiple publicly available datasets, including the National Database of Health Insurance Claims and Specific Health Checkups of Japan (NDB) Open Data. The NDB is a comprehensive database encompassing most of the data related to Japan's National Health Insurance system. Therefore, the representativeness of the NDB data is considered to be very high [17].

The NDB Open Data, accessible to researchers and the general public since fiscal year 2014, aggregates the number of medical and dental treatments by prefecture in the NDB data, excluding individual identification IDs. Notably, the inclusion of dental treatment data, which is a focus of this study, began in the fiscal year 2016. The NDB Open data is accessible to everyone free of charge and is actually used for several studies [18–20]. To align the survey timings for outcome and independent variables, a two-wave panel dataset was constructed for 2016 (wave 1) and 2018 (wave 2).

For more information about the data we used, please refer to the DATA AVAILABILITY STATEMENT section.

### Dependent variables

We used two dependent variables to assess dental care utilization and the oral health status of Japanese children from multiple perspectives: the standardized claim ratio (SCR) of deciduous tooth extractions (medical practice code number: 310000110) and the mean number of dental caries in 12-year-old children indicated by decayed, missing, or filled permanent teeth (DMFT).

Extractions of deciduous tooth were used as a proxy for dental visits for treatment. To determine the dental care utilization for children by prefecture, we used the 2016 and 2018 NDB Open Data and calculated SCRs. SCR is commonly used in research focusing on regional differences in medical care [18, 19]. It is an index of the level of dental care utilization relative to the value of 100 for Japan as a whole, indirectly adjusted for sex and age of the target prefecture. An SCR score higher than 100 means the prefecture utlilizes specific medical/dental care more frequently than the mean of Japan as a whole. For detailed SCR calculations, please refer to previous studies [19, 20]. The information on treatment for dental caries in children such as fillings, prosthetics, and dental pulp extractions was unavailable due to the following two reasons: First, the NDB Open data does not provide information on medical/dental claims by prefecture for specific age groups. Second, these data could not be differentiated by age because the rates for these dental services are the same for children and adults. We addressed this issue by focusing on dental practices that are mostly performed on children.

Another indicator of oral health at the community level were the DMFT scores for 12-year-olds acquired from school health statistics research conducted by the Ministry of Education, Culture, Sports, Science and Technology in 2016 and 2018. The DMFT scores have been commonly used in previous studies as a representative indicator of the oral health of children [10, 21].

### Independent variables

We used four independent variables: S-FMR program, medical-dental expense subsidy policies, SCR of dental sealants, and the per capita prefectural income. All independent variables were used from the survey data from 2016 and 2018.

The information about S-FMR in 2016 was provided by the Non-profit Japanese Conference on the Promotion of the Use of Fluoride in Caries Prevention. The information about S-FMR in 2018 was provided by the Ministry of Health, Labor and Welfare (MHLW). We used the survey data that included the number and percentage of institutions' students that used S-FMR at their facilities (i.e., nurseries, kindergartens, elementary schools, and junior high schools).

Each prefecture in Japan provides medical/dental expense assistance status for infants and young children in addition to the universal health insurance system (the eligible age varies in each prefecture). There are two types of subsidies: one exempts the full amount of the co-payment, and the other subsidizes the co-payment to a lower amount. The medical-dental expense subsidy policy information was used from other open data provided by the MHLW [22]. The MHLW conducted a survey to determine the medical/dental expense assistance status for infants and young children in each prefecture. The survey data included the eligibility age for deductible medical expenses and co-payments. From this data, we categorized the status of outpatient medical assistance in the following four categories: 1. Co-payment until children enter elementary school; 2. No co-payment until children enter elementary school; 3. Co-payment continues after children enter elementary school (till 18 years old); 4. No co-payment continues after children enter elementary school (till 18 years old). The co-payment in this case varies in each prefecture but does not exceed the co-payment amount for adults (30%). Since the number of prefectures opting for option 4 is small (for example, 4 out of 47 prefectures in 2018 data), options 3 and 4 were combined and three categories were created: 1. Co-payment until children enter elementary school; 2. No co-payment until children enter elementary school; and 3. Co-payment continuing beyond elementary school.

The SCR of dental sealants (medical practice code number: 309001710) was obtained from the NDB Open data as a proxy for dental visits for prevention.

Per capita prefectural income data was obtained from the Prefectural Accounts, key economic indicators used for prefectural policy-making. The Prefectural Accounts are formulated following the System of National Accounts and adhere to international standards established by the United Nations, and Japan's Statistics Law.

### Covariates

To examine other factors contributing to inequality among prefectures, the following variables published by the Japanese government were used: the number of dentists working in private offices per 10000 people, the unemployment rate (labor force survey), the rate of industrial structure 3 (service/commerce), percentage of college graduates, number of families per household, percentage of the nuclear family, the total fertility rate, the percentage of households living with older adults, and the percentage of receiving public assistance. These variables were not surveyed in 2016 and 2018, so the values published from the 2010 and 2015 surveys were used.

### Statistical analyses

First, we conducted descriptive statistics to clarify the relationships between dependent variables, independent variables, and covariates. We conducted Pearson's correlation test for continuous variables and calculated the correlation coefficients. In the case of continuous and categorical variables, we performed a one-way analysis of variance (ANOVA) to compute the F-values.

Next, multilevel mixed-effects linear regression analysis was performed for each dependent variable to clarify the association between the independent and dependent variables. Coefficient and 95% confidence intervals (95%CI) were calculated to facilitate the interpretation of the results of the linear regression analysis. Our analyses were conducted for five models: Model 1 includes the S-FMR and covariates, Model 2 includes the subsidy policy and covariates, Model 3 includes SCR of dental sealants and covariates, Model 4 includes per capita prefectural income and covariates, Model 5 includes Model 1 and the subsidy policy, SCR of dental sealants, and per capita prefectural income. Additionally, multilevel mixed-effects linear regression analysis was performed to assess the interaction effects to determine whether there are interaction effects between S-FMR, subsidy policies, the prevalence of dental sealants, and regional economic conditions. We analyzed these through six distinct models, each incorporating interaction terms alongside all independent variables and covariates. Interaction terms were: Model 1, subsidy policy and S-FMR; Model 2, subsidy policy and dental sealants; Model 3, subsidy policy and prefectural income; Model 4, S-FMR and dental sealants; Model 5, S-FMR and prefectural income; and Model 6, dental sealants and prefectural income.

All covariates were treated as continuous values and included in the model. Since the NDB Open data does not include medical claims data for public assistance recipients, the percentage of receiving public assistance was excluded from the model when analyzing the SCR of deciduous tooth extraction as the dependent variable. However, since the percentage of receiving public assistance can be considered a proxy indicator for SES at the community level, we included the percentage of receiving public assistance in the model conducted as a sensitivity analysis.

All analyses were performed using Stata (version 16.1; StataCorp LP, College Station, Texas). The threshold for significance was set at *P* < 0.05, 2-tailed.

## Results

**Fig. 1:**
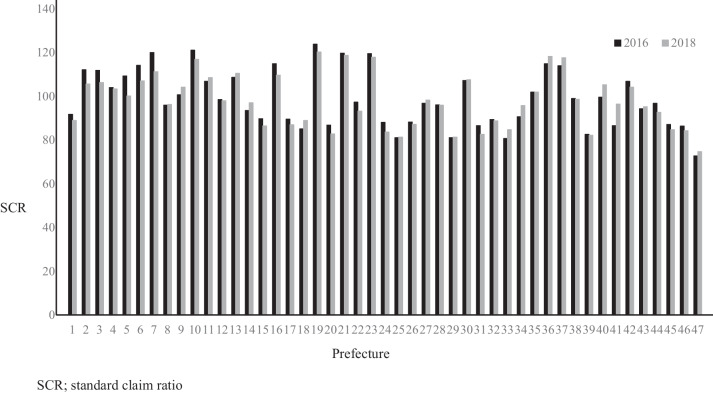
The SCR of deciduous tooth extraction trend among prefectures in Japan between 2016 to 2018. Legends: A figure shows the SCR of deciduous tooth extraction trends in each prefecture between 2016 and 2018

**Fig. 2:**
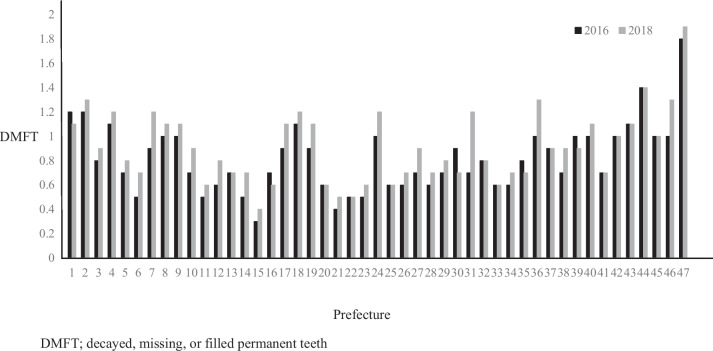
The DMFT score among prefectures in Japan between 2016 to 2018. Legends: A figure shows the DMFT score trends in each prefecture between 2016 and 2018

Table 1 presents the descriptive statistics for the data from all 47 prefectures from 2016 (wave 1) and 2018(wave 2). The mean SCR of deciduous tooth extractions was 98.8 (standard deviation (SD) = 12.9) in wave 1 and 97.9 (SD = 12.1) in wave 2. The DMFT for 12-year-olds was 0.9 (SD = 0.3) in wave 1 and 0.8 (SD = 0.3) in wave 2. The mean S-FMR utilization rate was 17.3% (SD = 21.1) in wave 1 and 19.4% (SD = 22.6) in wave 2. The mean SCR of dental sealants was 88.8 (SD = 36.6) in wave 1 and 88.0 (SD = 38.7) in wave 2. The mean Per capita prefectural income (1000 JPY) was 2894.7 (SD = 472.4) in wave 1 and 3003.8(SD = 467.9) in wave 2. The number of prefectures that provided subsidy policies were as follows: co-payment until the child enters elementary school (*n* = 25 in wave 1, *n* = 23 in wave 2), no co-payment until the child enters elementary school (*n* = 7 in wave 1, *n* = 7 in wave 2), and continuing the subsidy policy beyond elementary school (*n* = 15 in wave 1, *n* = 17 in wave 2). From the results of correlation analysis and ANOVA, the variables significantly associated with the SCR of deciduous tooth extraction were: SCR of dental sealants in wave 1 (*r* = 0.37), medical-dental expense subsidy policies in wave 1 (F = 5.59), SCR of dental sealants in wave 2 (*r* = 0.42), per capita prefectural income in wave 2 (*r* = 0.37), number of dentists in private dental offices in wave 2 (*r* = 0.33), and medical-dental expense subsidy policies in wave 2 (F = 5.57). Similarly, variables significantly associated with DMFT were: S-FMR utilization rate in wave 1 (*r* = -0.29), SCR of dental sealants in wave 1 (*r* = -0.34), per capita prefectural income in wave 1 (*r* = -0.32), the total fertility rate in wave 1 (*r* = 0.32), percentage of college graduates in wave 1 (*r* = -0.31), the unemployment rate in wave 1 (*r* = 0.44), the percentage receiving public assistance in wave 1 (*r* = 0.58), the total fertility rate in wave 2 (*r* = 0.40), and percentage receiving public assistance in wave 2 (*r* = 0.39). For more information about time series changes in each prefecture's dependent or independent variables, please refer to the figures (Figs. 1, 2, and Supplementary Figs. 1, 2, 3, 4).

**Table 1:** Descriptive statistics of the standardized claim ratio of deciduous tooth treatment and the number of decayed, missing, and filled permanent teeth for 12-year-olds in Japan (47 prefectures) from 2016 and 2018

	WAVE 1 (2016)						WAVE 2 (2018)					
					SCR of deciduous tooth extraction	DMFT					SCR of deciduous tooth extraction	DMFT
Continuous variable	Mean	SD	Min	Max	Correlation	Correlation	Mean	SD	Min	Max	Correlation	Correlation
SCR of deciduous tooth extraction	98.8	12.9	72.7	123.8			97.9	12.1	74.7	120.2		
DMFT for 12-year-olds (number)	0.9	0.3	0.4	1.9			0.8	0.3	0.3	1.8		
The rate of S-FMR utilization (per person)	17.3	21.1	0.1	86.9	-0.10	-0.29*	19.4	22.6	0.0	84.7	-0.08	-0.06
SCR of dental sealants	88.8	36.6	17.4	210.0	0.37*	-0.34*	88.0	38.7	27.1	230.4	0.42*	-0.28
Per capita prefectural income (1,000 JPY)	2894.7	472.4	2313.1	5400.3	0.29	-0.32*	3003.8	467.9	2391	5414.8	0.37*	-0.30
The rate of Industrial Structure 3 (Service / Commerce) (%) †	65.1	4.0	58.6	72.7	-0.27	0.23	68.7	5.0	61.5	82.1	-0.21	0.24
Number of families (per household) †	2.5	0.2	2.0	2.9	0.12	-0.05	2.4	0.2	2.0	2.8	0.03	-0.16
Percentage of the nuclear family (%) †	56.3	3.4	48.2	64.0	-0.21	0.02	56.1	3.1	47.8	63.9	-0.14	-0.02
Percentage of households living with older adults (%) †	41.5	5.5	28.8	53.1	0.18	-0.10	44.8	5.3	30.9	55.8	0.06	-0.13
Total fertility rate (%) †	1.5	0.1	1.1	1.9	-0.27	0.32*	1.5	0.1	1.2	2.0	-0.27	0.40*
Percentage of college graduates (%) †	16.5	5.3	9.4	34.7	-0.07	-0.31*	16.5	5.3	9.4	34.7	0.06	-0.35*
The unemployment rate (%) †	4.8	0.8	3.2	7.6	-0.05	0.44*	2.1	0.5	1.3	3.4	-0.09	0.31:
The number of dentists working in private dental office (per 100,000 people) †	69.6	13.1	50.6	118.7	0.15	-0.15	73.6	13.1	54.9	115.9	0.33*	-0.18:
Percentage of receiving public assistance (%) †	0.7	0.4	0.1	2.0	-0.10	0.58*	1.4	0.7	0.3	3.2	-0.18	0.39*
Categorical variable	Number of prefectures (Total *N* = 47)				F value	F value	Number of prefectures Total *N* = 47)				F value	F value
Medical-dental expense subsidy policies					5.59*	0.58					5.57*	0.63
Co-payment until children enter elementary school	25						23					
No co-payment until children enter elementary school	7						7					
Co-payment continuing beyond elementary school	15						17					

The relationship between the outcome variable and each variable was described, and further statistical tests were conducted to demonstrate their association

In each wave, variables marked with an “†” were not surveyed in the respective year due to the nature of the investigation, so values from other years (Wave 1 from 2010, Wave 2 from 2015) were substituted

Correlation coefficients were calculated using Pearson's correlation test, and the F values were derived from one-way analysis of variance (ANOVA)

*Abbreviations*: *SD* standard deviation, *SCR* standardized claim ratio, *DMFT* decayed, missing, or filled permanent teeth, *S-FMR* school-based fluoride mouth-rinse, *JPY* Japanese yen

^*^It means *P* < 0.05

Table 2 shows the associations of S-FMR and the subsidy policy on the SCR of deciduous tooth extractions. The results from Models 1 to 4 were largely consistent with the findings of Model 5. Therefore, we presented the results of Models 5 here. In Model 5, included Model 1 (S-FMR and covariates) and the subsidy policy, SCR of dental sealants, and per capita prefectural income. S-FMR was negatively associated with the SCR of deciduous tooth extractions (coefficient = -0.13, 95% CI -0.24; -0.02). No co-payment until the child enters elementary school was positively associated with the SCR of deciduous tooth extractions compared to co-payment until the child enters elementary school (coefficient = 11.42, 95% CI 3.29; 19.55). SCR of dental sealants was positively associated with the SCR of deciduous tooth extractions (coefficient = 0.12, 95% CI 0.06; 0.19). Per capita prefectural income was positively associated with the SCR of deciduous tooth extractions (coefficient = 0.01, 95% CI 0.001; 0.02).

**Table 2:** The associations between S-FMR and medical-dental expense subsidy policy on the SCR of deciduous tooth extraction

		Model 1		Model 2		Model 3		Model 4		Model 5	
		Coef. ^a^	95%CI	Coef. ^a^	95%CI	Coef. ^a^	95%CI	Coef. ^a^	95%CI	Coef. ^a^	95%CI
The rate of S-FMR utilization (per person) (%)		-0.13	(-0.24;-0.02)*							-0.11	(-0.20;-0.01)*
Subsidy policy (REF: Co-pay until children enters elementary school)	No co-payment until children enters elementary school			16.44	(7.75;25.14)*					11.42	(3.29;19.55)*
	Co-payment continuing beyond elementary school			4.24	(0.06;8.41)*					2.24	(-1.60;6.08)
SCR of dental sealants						0.15	(0.08;0.22)*			0.12	(0.06;0.19)*
Per capita prefectural income (1,000 JPY)								0.01	(0.003;0.02)*	0.01	(0.001;0.02)*
The rate of Industrial Structure 3 (Service / Commerce) (%)		0.27	(-0.23;0.77)	0.41	(-0.08;0.90)	0.37	(-0.10;0.84)	0.51	(-0.02;1.04)*	0.52	(0.06;0.98)*
Number of families (per household)		22.39	(1.64;43.13)*	17.17	(-2.58;36.92)	21.06	(1.37;40.76)	27.83	(7.49;48.16)*	15.56	(-2.54;33.66)
Percentage of the nuclear family (%)		-0.29	(-1.19;0.61)	-0.22	(-1.06;0.63)	-0.3	(-1.16;0.55)	0.17	(-0.73;1.07)	-0.27	(-1.07;0.53)
Percentage of households living with older adults (%)		0.38	(-0.35;1.10)	0.32	(-0.39;1.03)	0.36	(-0.33;1.05)	0.24	(-0.49;0.97)	0.41	(-0.24;1.05)
Total fertility rate (%)		-10.49	(-34.16;13.19)	-14.14	(-36.53;8.24)	-7.69	(-30.22;14.84)*	-11.59	(-34.87;11.70)	-11.56	(-31.69;8.57)
Percentage of college graduates (%)		-0.03	(-0.98;0.93)	-0.21	(-1.10;0.68)	0.06	(-0.85;0.96)	-0.65	(-1.71;0.40)	-0.77	(-1.68;0.15)
The unemployment rate (%)		0.24	(-1.23;1.72)	0.72	(-0.76;2.19)	0.44	(-0.96;1.85)	0.67	(-0.86;2.20)	1.03	(-0.31;2.38)
The number of dentists working in private dental office (per 100,000 people)		0.21	(-0.03;0.45)	0.23	(0.005;0.46)*	0.14	(-0.10;0.37)	0.19	(-0.05;0.43)	0.09	(-0.13;0.30)

Model 1: S-FMR + covariates (industrial structure 3 rate, number of families, percentage of nuclear family, percentage of households living with older adults, total fertility rate, percentage of college graduates, unemployment rate, total fertility rate, and dentist in private practice); Model 2: policy + covariates; Model 3: SCR of dental sealants + covariates; Model 4: per capita prefectural income + covariates; Model 5: Model 1 + policy + SCR of dental sealants + per capita prefectural income

The analysis was conducted to identify predictors for the SCR of deciduous tooth extraction

*Abbreviations*: *Coef.* Coefficient, *SCR* standardized claim ratio, *S-FMR* school-based fluoride mouth-rinse, *CI* confidence interval, *REF* reference

^*^ It means *P* < 0.05

^a^Coefficient was estimated using a multilevel mixed-effects linear regression model with all variables simultaneously entered into the model

Table 3 shows the Interaction of each independent variable in the SCR of deciduous tooth extraction. Among the interaction terms, only in Model 4 was a significant association observed. In Model 4, the interaction terms between S-FMR and SCR of dental sealants were positively associated with the SCR of deciduous tooth extraction (coefficient = 0.004, 95% CI 0.001; 0.01). In this model, S-FMR was negatively associated with the SCR of deciduous tooth extractions (coefficient = -0.46, 95% CI -0.70; -0.21, while dental sealants were not associated with the SCR of deciduous tooth extractions (coefficient = 0.04, 95% CI -0.04; 0.13).

**Table 3:** Interaction of each independent variable in the SCR of deciduous tooth extraction

		Model 1		Model 2		Model 3		Model 4		Model 5		Model 6	
		Coef.^a^	95%CI	Coef.^a^	95%CI	Coef.^a^	95%CI	Coef.^a^	95%CI	Coef.^a^	95%CI	Coef.^a^	95%CI
	The rate of S-FMR utilization (per person) (%)	-0.10	(-0.21; 0.003)	-0.11	(-0.20; -0.01)*	-0.104	(-0.200; -0.008)*	-0.46	(-0.70; -0.21)*	-0.63	(-1.32; 0.05)	-0.11	(-0.20; -0.01)*
Subsidy policy (REF: Co-pay until children enters elementary school)	No co-payment until children enters elementary school	8.70	(-3.3; 20.71)	12.34	(-3.91; 28.58)	51.12	(1.15; 101.09)*	11.45	(3.47; 19.44)*	11.13	(2.84; 19.42)*	11.42	(3.12; 19.71)*
	Co-payment continuing beyond elementary school	2.56	(-1.5; 6.62)	-10.37	(-24.14; 3.39)	15.72	(-13.73; 45.16)	2.79	(-0.85; 6.43)	2.56	(-1.21; 6.32)	2.00	(-1.76; 5.75)
	SCR of dental sealants	0.12	(0.05; 0.19)*	0.11	(0.01; 0.21)	0.13	(0.07; 0.20)*	0.04	(-0.04; 0.13)	0.12	(0.05; 0.19)*	0.40	(0.03; 0.77)*
	Per capita prefectural income (1,000 JPY)	0.01	(0.0003; 0.02)*	0.01	(0.002; 0.017)*	0.010	(0.002; 0.024)*	0.01	(-0.001; 0.014)	0.01	(-0.002; 0.014)	0.020	(0.003; 0.029)*
	The rate of Industrial Structure 3 (Service / Commerce) (%)	0.52	(0.05; 0.98)*	0.50	(0.05; 0.96)*	0.49	(0.03; 0.96)*	0.53	(0.09; 0.96)*	0.58	(0.12; 1.03)*	0.450	(-0.008; 0.914)
	Number of families (per household)	14.59	(-4.50; 33.69)	16.17	(-1.75; 34.1)	14.63	(-3.23; 32.50)	14.26	(-3.21; 31.73)	18.38	(0.07; 36.68)*	14.42	(-3.75; 32.58)
	Percentage of the nuclear family (%)	-0.34	(-1.17; 0.49)	-0.30	(-1.09; 0.49)	-0.26	(-1.05; 0.53)	-0.31	(-1.08; 0.46)	-0.37	(-1.17; 0.43)	-0.41	(-1.22; 0.40)
	Percentage of households living with older adults (%)	0.39	(-0.26; 1.04)	0.46	(-0.17; 1.09)	0.36	(-0.29; 1.01)	0.47	(-0.15; 1.08)	0.36	(-0.28; 0.99)	0.41	(-0.22; 1.05)
	Total fertility rate (%)	-13.21	(-33.83; 7.41)	-10.84	(-30.87; 9.18)	-8.77	(-29.12; 11.57)	-14.7	(-34.23; 4.84)	-10.44	(-30.49; 9.62)	-7.74	(-28.25; 12.76)
	Percentage of college graduates (%)	-0.79	(-1.71; 0.14)	-0.69	(-1.60; 0.21)	-0.83	(-1.74; 0.09)	-0.70	(-1.59; 0.19)	-0.72	(-1.64; 0.20)	-0.65	(-1.58; 0.28)
	The unemployment rate (%)	1.03	(-0.32; 2.38)	1.03	(-0.29; 2.35)	1.11	(-0.22; 2.44)	1.11	(-0.17; 2.39)	1.089	(-0.23; 2.41)	1.05	(-0.26; 2.37)
	The number of dentists working in private dental office (per 100,000 people)	0.08	(-0.14; 0.30)	0.05	(-0.17; 0.26)	0.09	(-0.12; 0.31)	0.12	(-0.08; 0.33)	0.13	(-0.09; 0.35)	0.07	(-0.14; 0.28)
Interaction term policy and S-FMR (REF: Co-pay until children enters elementary school * S-FMR)	No co-payment until children enters elementary school * S-FMR	0.21	(-0.45; 0.87)										
	Co-payment continuing beyond elementary school * S-FMR	-0.04	(-0.25; 0.17)										
Interaction term policy and dental sealant (REF: Co-pay until children enters elementary school * SCR of dental sealants)	No co-payment until children enters elementary school * S-FMR			-0.02	(-0.16; 0.13)								
	Co-payment continuing beyond elementary school * S-FMR			0.12	(-0.01; 0.24)								
Interaction term policy and prefectural income (REF: Co-pay until children enters elementary school * Per capita prefectural income)	No co-payment until children enters elementary school * S-FMR					-0.013	(-0.03; 0.003)						
	Co-payment continuing beyond elementary school * S-FMR					-0.005	(-0.015; 0.006)						
Interaction term S-FMR and SCR of dental sealants								0.004	(0.001;0.01)*				
Interaction term S-FMR and Per capita prefectural income										0.0002	(-0.0001; 0.0004)		
Interaction term SCR of dental sealants and per capita prefectural income												-0.00009	(-0.0002; 0.00003)

Each model incorporates different interaction terms. The interaction terms for each model are as follows: Model 1; policy and S-FMR, Model 2; policy and dental sealant, Model 3; policy and prefectural income, Model 4; S-FMR and SCR of dental sealants, Model 5; S-FMR and per capita prefectural income, Model 6; SCR of dental sealants and per capita prefectural income

The analysis was conducted to examine the presence of six types of interaction terms on the SCR of deciduous tooth extraction

*Abbreviations*: *Coef.* Coefficient, *SCR* standardized claim ratio, *S-FMR* school-based fluoride mouth-rinse, *CI* confidence interval, *REF* reference

^*^It means *P* < 0.05

^a^Coefficient was estimated using a multilevel mixed-effects linear regression model with all variables simultaneously entered into the model

Table 4 shows the associations of S-FMR and the subsidy policy on the DMFT. The results from Models 1 to 4 were largely consistent with the findings of Model 5. Therefore, we presented the results of Models 5 here. In Model 5, included Model 1(S-FMR and covariates) and the subsidy policy, SCR of dental sealants, and per capita prefectural income. S-FMR was negatively associated with the SCR of deciduous tooth extractions coefficient = -0.003, 95% CI -0.005; -0.001). SCR of dental sealants was negatively associated with DMFT (coefficient = -0.0013, 95% CI -0.0025; -0.0001).

**Table 4:** The associations between S-FMR and medical-dental expense subsidy policy on children's DMFT

		Model 1		Model 2		Model 3		Model 4		Model 5	
		Coef.^a^	95%CI	Coef.^a^	95%CI	Coef.^a^	95%CI	Coef.^a^	95%CI	Coef.^a^	95%CI
The rate of S-FMR utilization (per person) (%)		-0.004	(-0.006; -0.001)*							-0.003	(-0.005; -0.001)*
Subsidy policy (REF: Co-pay until children enters elementary school)	No co-payment until children enters elementary school			0.02	(-0.13;0.18)					0.05	(-0.08;0.18)
	Co-payment continuing beyond elementary school			0.09	(-0.01;0.20)					0.07	(-0.02;0.17)
SCR of dental sealants						-0.001	(-0.003;-0.0001)*			-0.001	(-0.003;-0.0001)*
Per capita prefectural income (1,000 JPY)								0.0001	(-0.0001;0.0003)	0.00004	(-0.0001;0.0002)
The rate of Industrial Structure 3 (Service / Commerce) (%)		0.01	(-0.004;0.022)	0.01	(-0.01;0.02)	0.005	(-0.01;0.02)	0.008	(-0.007;0.02)	0.01	(-0.002;0.03)
Number of families (per household)		-0.16	(-0.55;0.23)	-0.19	(-0.60;0.23)	-0.09	(-0.50;0.32)	-0.13	(-0.56;0.30)	-0.12	(-0.50;0.26)
Percentage of the nuclear family (%)		-0.01	(-0.02;0.007)	-0.004	(-0.02;0.01)	-0.004	(-0.02;0.01)	-0.002	(-0.022;0.02)	-0.004	(-0.02;0.01)
Percentage of households living with older adults (%)		-0.02	(-0.03; -0.001)	-0.02	(-0.04; -0.01)*	-0.02	(-0.04; -0.01)*	-0.02	(-0.04; -0.002)*	-0.02	(-0.03; -0.003)*
Total fertility rate (%)		0.45	(0.01;0.88)	0.44	(-0.03;0.90)	0.36	(-0.10;0.82)	0.41	(-0.07;0.88)	0.46	(0.05;0.87)*
Percentage of college graduates (%)		-0.03	(-0.05; -0.02)*	-0.03	(-0.05; -0.02)*	-0.03	(-0.05; -0.01)*	-0.03	(-0.05;-0.01)*	-0.04	(-0.06; -0.02)*
Percentage of receiving public assistance (%)		0.10	(0.04;0.17)*	0.10	(0.03;0.17)*	0.11	(0.04;0.17)*	0.11	(0.04;0.17)*	0.10	(0.04;0.17)*
The unemployment rate (%)		0.06	(0.03;0.10)*	0.06	(0.02;0.10)*	0.05	(0.02;0.09)*	0.06	(0.02;0.10)*	0.07	(0.03;0.10)*
The number of dentists working in private dental office (per 100,000 people)		-0.004	(-0.009; -0.0001)*	-0.004	(-0.01;0.0004)	-0.003	(-0.01;0.002)	-0.005	(-0.01;0.0003)	-0.003	(-0.008;0.001)

Model 1: S-FMR + covariates (industrial structure 3 rate, number of families, percentage of nuclear family, percentage of households living with older adults, total fertility rate, percentage of college graduates, percentage of receiving public assistance, unemployment rate, total fertility rate, and dentist in private practice); Model 2: policy + covariates; Model 3: SCR of dental sealants + covariates; Model 4: per capita prefectural income + covariates; Model 5: Model 1 + policy + SCR of dental sealants + per capita prefectural income

The analysis was conducted to identify predictors for DMFT

*Abbreviations*: *Coef.* Coefficient, *DMFT* decayed, missing, or filled permanent teeth, *S-FMR* school-based fluoride mouth-rinse, *SCR* standardized claim ratio, *CI* confidence interval, *REF* reference

^*^It means *P* < 0.05

^a^Coefficient was estimated using a multilevel mixed-effects linear regression model with all variables simultaneously entered into the model

Table 5 shows the Interaction of each independent variable in children's DMFT. Among the interaction terms, Model 2 and Model 4 were significant associations observed. In Model 2, the interaction terms between subsidy policy and SCR of dental sealants were negatively associated with DMFT (coefficient = -0.003, 95% CI -0.006; -0.0005). In this model, the subsidy policy and SCR of dental sealants were not associated with DMFT. In Model 4, the interaction terms between S-FMR and SCR of dental sealants were positively associated with DMFT (coefficient = 0.0001, 95% CI 0.00003; 0.0002). In this model, S-FMR and SCR of dental sealants were negatively associated with DMFT (coefficient = -0.012, 95% CI -0.018; -0.005, coefficient = -0.003, 95% CI -0.005; -0.002, respectively).

**Table 5:** Interaction of each independent variable in children's DMFT

		Model 1		Model 2		Model 3		Model 4		Model 5		Model 6	
		Coef.^a^	95%CI	Coef.^a^	95%CI	Coef.^a^	95%CI	Coef.^a^	95%CI	Coef.^a^	95%CI	Coef.^a^	95%CI
	The rate of S-FMR utilization (per person) (%)	-0.002	(-0.047: 0.0001)	-0.003	(-0.005; -0.001)*	-0.003	(-0.005; -0.001)*	-0.012	(-0.018; -0.005)*	-0.005	(-0.024; 0.015)	-0.003	(-0.005; -0.001)*
Subsidy policy (REF: Co-pay until children enters elementary school)	No co-payment until children enters elementary school	0.079	(-0.150; 0.307)	0.13	(-0.13; 0.39)	0.16	(-1.20; 1.51)	0.05	(-0.08; 0.17)	0.05	(-0.09; 0.18)	0.05	(-0.08; 0.18)
	Co-payment continuing beyond elementary school	0.114	(-0.003; 0.230)	0.38	(0.11; 0.65)*	-0.26	(-1.01; 0.49)	0.088	(-0.005; 0.182)	0.08	(-0.02; 0.17)	0.08	(-0.02; 0.18)
	SCR of dental sealants	-0.001	(-0.003; -0.00003)*	-0.0005	(-0.0020; 0.0010)	-0.00120	(-0.00244; 0.00006)	-0.0033	(-0.005; -0.002)*	-0.0014	(-0.0026; -0.0001)*	-0.003	(-0.013; 0.007)
	Per capita prefectural income (1,000 JPY)	0.00002	(-0.00014; 0.00019)	0.00003	(-0.00012; 0.00018)	-0.00005	(-0.00033; 0.00023)	0.00001	(-0.00014; 0.00017)	0.00004	(-0.00013; 0.00021)	0.000001	(-0.000345; 0.000348)
	The rate of Industrial Structure 3 (Service / Commerce) (%)	0.012	(-0.003; -0.00003)	0.012	(-0.001; 0.026)	0.011	(-0.004; 0.025)	0.012	(-0.001; 0.026)	0.012	(-0.002; 0.026)	0.012	(-0.002; 0.027)
	Number of families (per household)	-0.189	(-0.0001; 0.0002)	-0.12	(-0.48; 0.24)	-0.12	(-0.49; 0.26)	-0.12	(-0.47; 0.24)	-0.12	(-0.50; 0.26)	-0.11	(-0.49; 0.27)
	Percentage of the nuclear family (%)	-0.006	(-0.002; 0.025)	-0.004	(-0.02; 0.012)	-0.004	(-0.02; 0.013)	-0.005	(-0.021; 0.01)	-0.004	(-0.020; 0.013)	-0.003	(-0.020; 0.014)
	Percentage of households living with older adults (%)	-0.017	(-0.578; 0.200)*	-0.018	(-0.033; -0.003)*	-0.017	(-0.033; -0.002)*	-0.016	(-0.031; -0.002)*	-0.018	(-0.033; -0.003)*	-0.018	(-0.033; -0.003)*
	Total fertility rate (%)	0.412	(-0.023; 0.011)	0.49	(0.11; 0.88)*	0.46	(0.04; 0.87)*	0.44	(0.06; 0.82)*	0.46	(0.05; 0.87)*	0.45	(0.03; 0.87)*
	Percentage of college graduates (%)	-0.04	(-0.032; -0.002)*	-0.04	(-0.06; -0.02)*	-0.04	(-0.05; -0.02)*	-0.04	(-0.05; -0.02)*	-0.04	(-0.06; -0.02)*	-0.04	(-0.06; -0.02)*
	Percentage of receiving public assistance (%)	0.103	(-0.015; 0.839)*	0.11	(0.04; 0.18)*	0.11	(0.04; 0.17)*	0.11	(0.04; 0.17)*	0.11	(0.04; 0.17)*	0.10	(0.03; 0.17)*
	The unemployment rate (%)	0.069	(-0.055; -0.020)*	0.07	(0.03; 0.11)*	0.06	(0.03; 0.10)*	0.07	(0.04; 0.11)*	0.07	(0.03; 0.11)*	0.07	(0.03; 0.11)*
	The number of dentists working in private dental office (per 100,000 people)	-0.004	(0.035; 0.171)	-0.003	(-0.007; 0.001)	-0.004	(-0.008; 0.001)	-0.002	(-0.006; 0.002)	-0.003	(-0.008; 0.001)	-0.003	(-0.008; 0.001)
Interaction term policy and S-FMR (REF: Co-pay until children enters elementary school * S-FMR)	No co-payment until children enters elementary school * S-FMR	-0.001	(0.032; 0.106)										
	Co-payment continuing beyond elementary school * S-FMR	-0.002	(-0.008; 0.0003)										
Interaction term policy and dental sealant (REF: Co-pay until children enters elementary school * SCR of dental sealants)	No co-payment until children enters elementary school * S-FMR			-0.0008	(-0.0032; 0.0016)								
	Co-payment continuing beyond elementary school * S-FMR			-0.0033	(-0.0061; -0.0005)*								
Interaction term policy and prefectural income (REF: Co-pay until children enters elementary school * Per capita prefectural income)	No co-payment until children enters elementary school * S-FMR					-0.00003	(-0.00048; 0.00042)						
	Co-payment continuing beyond elementary school * S-FMR					0.0001	(-0.00014; 0.00037)						
Interaction term S-FMR and SCR of dental sealants								0.0001	(0.00003;0.0002)*				
Interaction term S-FMR and Per capita prefectural income										0.000001	(-0.00001; 0.00001)		
Interaction term SCR of dental sealants and per capita prefectural income												0.0000004	(-0.0000027; 0.0000035)

Each model incorporates different interaction terms. The interaction terms for each model are as follows: Model 1; policy and S-FMR, Model 2; policy and dental sealant, Model 3; policy and prefectural income, Model 4; S-FMR and SCR of dental sealants, Model 5; S-FMR and per capita prefectural income, Model 6; SCR of dental sealants and per capita prefectural income

The analysis was conducted to examine the presence of six types of interaction terms on children's DMFT

*Abbreviations*: *Coef.* Coefficient, *SCR* standardized claim ratio, *S-FMR* school-based fluoride mouth-rinse, *CI* confidence interval, *REF* reference

^*^It means *P* < 0.05

^a^Coefficient was estimated using a multilevel mixed-effects linear regression model with all variables simultaneously entered into the model

The sensitivity analysis results that included the percentage of public assistance in each prefecture as a proxy for community-level SES in the model were almost the same as the results in Table 2 (shown in Supplementary Table 1).

## Discussion

Our study found that children's oral health was associated with S-FMR, subsidy policy, dental sealants, and prefectural income. Additionally, we assessed for interaction effects to determine whether there are interaction effects between S-FMR, subsidy policies, the prevalence of dental sealants, and regional economic conditions. Two interaction terms, S-FMR with dental sealants and the subsidy policy with dental sealants, showed significant associations with children's oral health, however, no interaction effect existed between S-FMR and the subsidy policy. Our results are consistent with previous studies reporting improved oral health measures with S-FMR [10, 23] and improved oral health measures with a decrease in the co-payment rate [24, 25].

S-FMR was negatively associated with the SCR of deciduous tooth extractions and DMFT, even after adjusting for the influence of the subsidy policy, dental sealants, and prefectural income. The subsidy policy may increase children’s dental care use and result in higher SCR for deciduous tooth extraction rates. This result reinforces that S-FMR is an effective universal approach to maintaining oral health in children [10].

A positive association between the subsidy policy and the SCR of deciduous tooth extractions was found. This finding was consistent with the previous studies that free out-of-pocket medical expenses encourage hospital visits [15, 26]. However, no association was found between the subsidy policy and DMFT. This suggests that the effect of the subsidy policy on DMFT may be limited.

The SCR of dental sealants was negatively associated with children’s poor oral health, a finding consistent with systematic review [5]. Interestingly, the point estimates for dental sealants remained largely unchanged before and after adjusting for S-FMR and other independent variables. This suggests that the impact of dental sealants on children's oral health may operate via an independent pathway.

The positive point estimates for prefectural income, associated with deciduous tooth extractions and DMFT, align with the results of a past critical review [27]. Wealthier communities are likely to have better access to dental clinics [28], which could lead to more frequent dental visits. Considering that extractions may result from regular dental visits and frequent clinical experiences, the deciduous tooth extractions observed in wealthier communities could be of healthy deciduous teeth during the tooth exchange period rather than extractions due to severe caries.

The interaction term between the subsidy policy and dental sealants demonstrated a significant negative association with DMFT, which could be interpreted as the subsidy policy encouraging the implementation of dental sealants. Additionally, there was no significant association between the interaction of S-FMR and the subsidy policy with DMFT or deciduous tooth extractions. This suggests that these interventions might operate through independent pathways. While the subsidy policy contributes to improved access to dental care, considering its association with an increase in deciduous tooth extractions, it might have a greater impact on treatment visits rather than prevention.

The point estimate for the interaction between S-FMR and dental sealants was small but positive. Considering the biological mechanisms where S-FMR primarily prevents smooth surface caries and dental sealants prevent pit and fissure caries, it is inappropriate to interpret that implementing both interventions would decrease caries prevention effectiveness. This suggests that the distinction between health policies and health systems is crucial for effective decision-making in oral health. Health policies, like S-FMR and water fluoridation, set the direction for oral health initiatives [29, 30], while health systems, which include services like dental sealants, provide the organizational structure for health care delivery [30, 31]. Our study's findings align with this distinction, indicating that prefectures with extensive dental sealant use may not be as engaged in promoting S-FMR (Supplementary Fig. 5). In countries other than Japan, a municipality has been observed switching from health policy approaches to implementing medical policies more focused on health systems [32]. Researchers and governments must continue informing residents that S-FMR and dental sealants are an equitable and beneficial approach to maintaining oral health [33].

One implication is offered from this study. Many health interventions have a limited impact on vulnerable populations, such as those with low SES or poor health status, which leads to "inverse care laws" [34, 35]. While the subsidy policy and dental sealants may have effects that apply to all populations, factors such as geographic access to clinics are also relevant to actual dental visits [36–38]. Thus, the health effects of the subsidy policy among vulnerable populations may be limited. Whereas, S-FMR could help vulnerable populations because it targets entire institutions, such as schools, and may reduce DMFT, one of the indicators of oral health at the community level. A previous study showed that children's oral health worsened in municipalities that discontinued water fluoridation and switched to providing a dental sealant program [39]. To protect children's oral health, policymakers need to consider comprehensively promoting S-FMR, implementing subsidy policies, and providing dental sealants.

The strength of this study is that the validity of the results is ensured as the data targeted the entire population of Japan. The study had some limitations. First, causal relationships are unknown because this ecological study used two-wave panel data. An ecological study can lead to the ecological fallacy that associations observed between variables at the aggregated level do not necessarily represent associations at the individual level [40]. However, this can be avoided when previous studies have shown causal relationships at the individual level [41], such as the association between subsidy policies and oral health [42], and S-FMR and oral health [10]. Therefore, the potential for ecological fallacy in this study is limited.

Second, due to the unavailability of detailed public data at the municipal level, our sample size was limited to 47 prefectures, which restricted the statistical power of cross-sectional data. To overcome this limitation, we created and analyzed a two-wave panel dataset. For the implementation of more effective medical policies, it is essential to prepare more detailed municipal-level data, collect data that could be potential confounders, and advance the openness of data.

Third, using SCR variables to indicate children's oral health in the community may not have been appropriate because the NDB Open data are only from patients who visited dentists. While the NDB database is highly representative because it includes data on all medical procedures, it does not include data on residents who do not use medical facilities. Therefore, reimbursement data is unlikely to accurately reflect local chronic disease prevalence rates for which residents have little awareness of the need for treatment. In fact, it has been found that medical visits are lower in areas with poor access to medical facilities [12, 43, 44]. Therefore, the present study addressed this issue by examining the association between S-FMR and the subsidy policy, including DMFT, a dependent variable, other than NDB Open data.

Finally, using DMFT as an indicator of children's oral health may not have been appropriate in identifying associations between the subsidy policy for children's oral health in the community because the subsidy policy may have decreased the number of D (untreated decayed teeth) and increased the number of F (filled teeth) among DMFT. Therefore, we conducted a sensitivity analysis using D, an indicator of untreated decayed teeth, as a dependent variable instead of DMFT. The results were similar to those in the main analysis (Supplementary Table 2). The subsidy policy does not appear to be associated with dental visits for prevention, at least in childhood.

## Conclusion

To maintain childhood oral health, policymakers need to promote not only the strengthening of health systems, such as support for dental visits for treatment but also the enhancement of health policies, like the implementation of S-FMR.

### Supplementary Information

**Supplementary Material 1.**

**Supplementary Material 2.**

**Supplementary Material 3.**

**Supplementary Material 4.**

**Supplementary Material 5.**

**Supplementary Material 6.**

## Data Availability

In this study, we used information for each variable from various URLs and combined them into a single data set. All the data sources are from Japanese government institutions, such as the Ministry of Health, Labour and Welfare, the Ministry of Education, Culture, Sports, Science and Technology, the Ministry of Economy, Trade and Industry, the Cabinet Office, as well as a non-profit organization. SCR of deciduous tooth extraction and SCR of dental sealants were calculated from NDB Open Data in 2016 (https://www.mhlw.go.jp/stf/seisakunitsuite/bunya/0000177221_00002.html) and 2018 (https://www.mhlw.go.jp/stf/seisakunitsuite/bunya/0000177221_00008.html). The DMFT scores for 12-year-olds was calculated from School Health Statistics Research in 2016 and 2018 (http://www.mext.go.jp/b_menu/toukei/chousa05/hoken/1268826.htm). S-FMR was used from surveys related to the promotion of dental and oral health, including caries control in 2016 (http://www.nponitif.jp/2016FMRarticle.pdf) and 2018 (https://www.mhlw.go.jp/stf/seisakunitsuite/bunya/kenkou_iryou/iryou/shika_hoken_jouhou/usyokutaisaku.html). Medical-dental expense subsidy policy was used from Survey on Medical expense support for infants in FY2016 (https://www.mhlw.go.jp/file/04-Houdouhappyou-11908000-Koyoukintoujidoukateikyoku-Boshihokenka/bessi2.pdf) and 2018 (https://www.mhlw.go.jp/stf/houdou/0000213116_00001.html). Per capita prefectural income data was obtained from the Prefectural Accounts, Cabinet Office in 2016 and 2018 (https://www.esri.cao.go.jp/jp/sna/data/data_list/kenmin/files/contents/main_2018.html). The ratio of dentists working in a dental private office was used from statistics of physicians, dentists and pharmacists in 2010 (https://www.mhlw.go.jp/toukei/saikin/hw/ishi/10/dl/toukeihyo.pdf) and 2015 (https://www.mhlw.go.jp/toukei/saikin/hw/ishi/18/index.html). The unemployment rate in 2010 (https://www.stat.go.jp/data/roudou/report/2010/index.html) and 2015 was used from the labor force survey (https://www.stat.go.jp/data/roudou/report/2015/index.html). The rate of industrial structure 3 (service/commerce), number of families (per household), the nuclear family rate, and percentage of households living with older adults were used from the population census in 2010 (https://www.e-stat.go.jp/stat-search/files?stat_infid=000025518704) and 2015 (https://www.e-stat.go.jp/stat-search/files?tclass=000001112558). The rate of college graduates in 2010 (https://www.e-stat.go.jp/stat-search/files?stat_infid=000031369054) and 2015 (https://www.e-stat.go.jp/stat-search/files?stat_infid=000028462319) was used from the social and population statistics system. The total fertility rate in 2010 (https://empowerment.tsuda.ac.jp/detail/12805) and 2015 (https://www.mhlw.go.jp/toukei/saikin/hw/jinkou/kakutei15/dl/07_h3-2.pdf) was used from Vital Statistics. The percentage of receiving public assistance in 2010 (https://www.e-stat.go.jp/stat-search/files?stat_infid=000012903232) and 2015 (https://www.e-stat.go.jp/stat-search/files?stat_infid=000031927623) was used from National Survey on public assistance recipients.

## References

[1] Tinanoff N, Baez RJ, Diaz Guillory C, Donly KJ, Feldens CA, McGrath C (2019). Early childhood caries epidemiology, aetiology, risk assessment, societal burden, management, education, and policy: global perspective. Int J Paediatr Dent.

[2] Heilmann A, Tsakos G, Watt R. Oral Health Over the Life Course, in a Life Course Perspective on Health Trajectories and Transitions. In: Life Course Research and Social Policies. Cham (CH): Springer; 2015. p. 39–59.

[3] Aida J, Matsuyama Y, Tabuchi T, Komazaki Y, Tsuboya T, Kato T (2017). Trajectory of social inequalities in the treatment of dental caries among preschool children in Japan. Community Dent Oral Epidemiol.

[4] Ran T, Chattopadhyay SK (2016). Economic evaluation of community water fluoridation: a community guide systematic review. Am J Prev Med.

[5] Ahovuo-Saloranta A, Forss H, Walsh T, Nordblad A, Mäkelä M, Worthington HV (2017). Pit and fissure sealants for preventing dental decay in permanent teeth. Cochrane Database Syst Rev..

[6] Iheozor-Ejiofor Z, O’Malley LA, Glenny AM, Macey R, Alam R, Tugwell P (2013). Water fluoridation for the prevention of dental caries. Cochrane Database Syst Rev.

[7] Centers for Disease Control and Prevention. Ten Great Public Health Achievements -- United States, 1900–1999 [Internet]. Centers for Disease Control and Prevention. 1999. Available from: https://www.cdc.gov/MMWR/preview/mmwrhtml/00056796.htm. Cited 2023 Feb 22. 10227303

[8] Matsuo G, Aida J, Osaka K, Rozier RG (2020). Effects of Community water fluoridation on dental caries disparities in adolescents. Int J Environ Res Public Health.

[9] Divaris K, Rozier RG, King RS (2012). Effectiveness of a school-based fluoride mouthrinse program. J Dent Res.

[10] Matsuyama Y, Aida J, Taura K, Kimoto K, Ando Y, Aoyama H (2016). School-based fluoride mouth-rinse program dissemination associated with decreasing dental caries inequalities between Japanese prefectures: an ecological study. J Epidemiol.

[11] Benzian H, Guarnizo-Herreño CC, Kearns C, Muriithi MW, Watt RG (2021). The WHO global strategy for oral health: an opportunity for bold action. Lancet.

[12] Wang TT, Mathur MR, Schmidt H (2020). Universal health coverage, oral health, equity and personal responsibility. Bull World Health Organ.

[13] National Academies of Sciences Engineering and Medicine. Crossing the global quality chasm: Improving health care worldwide. The National Acedemies Press. Washington, DC: The National Academies Press; 2018. 203–225 p. Available from: https://www.ncbi.nlm.nih.gov/books/NBK535653/pdf/Bookshelf_NBK535653.pdf. 30605296

[14] Aida J, Fukai K, Watt RG (2021). Global Neglect of dental coverage in Universal health coverage systems and japan’s broad coverage. Int Dent J.

[15] Iizuka T, Shigeoka H (2022). Is zero a special price? Evidence from child health care. Am Econ J Appl Econ.

[16] Matsuyama Y, Tsuboya T, Bessho SI, Aida J, Osaka K (2018). Copayment exemption policy and healthcare utilization after the great east Japan earthquake. Tohoku J Exp Med.

[17] Ministry of Health L and W. The 1st NDB Open Data Commentary (in Japanese). 2016. Available from: https://www.mhlw.go.jp/file/06-Seisakujouhou-12400000-Hokenkyoku/0000141549.pdf. Access 7 Dec 2023.

[18] Tanaka H, Onoda T, Ishii T (2022). Understanding the actual use of anti-HIV drugs in Japan from 2016 to 2019: demonstrating epidemiological relevance of NDB open data Japan for understanding Japanese medical care. Int J Environ Res Public Health.

[19] Taira K, Mori T, Ishimaru M, Iwagami M, Sakata N, Watanabe T (2021). regional inequality in dental care utilization in Japan: an ecological study using the national database of health insurance claims. Lancet Reg Heal - West Pacific.

[20] Kodama T, Ida Y, Oshima K, Miura H (2021). Are public oral care services evenly distributed?—nation-wide assessment of the provision of oral care in japan using the national database of health insurance claims. Int J Environ Res Public Health.

[21] Ohara S, Kawaguchi Y, Shinada K, Sasaki Y (2000). Evaluation of school-based dental health activities including fluoride mouth-rinsing in Hiraizumi. Japan J Med Dent Sci.

[22] Ministry of Health L and W. Survey on Medical expense support for infants in FY2018 (in Japanese). Ministry of Health, Labour and Welfare. 2019. Available from: https://www.mhlw.go.jp/stf/houdou/0000213116_00001.html. Cited 2023 Feb 22.

[23] Divaris K, Rozier RG, King RS (2012). Effectiveness of a school-based fluoride mouthrinse program. J Dent Res.

[24] Cooray U, Aida J, Watt RG, Tsakos G, Heilmann A, Kato H, et al. Effect of Copayment on Dental Visits: A Regression Discontinuity Analysis. J Dent Res. 2020 Nov 1;99(12):1356–62. Available from: https://pubmed.ncbi.nlm.nih.gov/32735476/. Cited 2021 Nov 22. 10.1177/0022034520946022 32735476

[25] Hoshi M, Aida J, Kusama T, Yamamoto T, Kiuchi S, Yamamoto T (2020). Is the association between green tea consumption and the number of remaining teeth affected by social networks?: A cross-sectional study from the Japan gerontological evaluation study project. Int J Environ Res Public Health.

[26] Du W, Liu P, Xu W (2022). Effects of decreasing the out-of-pocket expenses for outpatient care on health-seeking behaviors, health outcomes and medical expenses of people with diabetes: evidence from China. Int J Equity Health.

[27] Singh A, Peres MA, Watt RG (2019). The relationship between income and oral health: a critical review. J Dent Res.

[28] Da Silva AN, Mendonça MH, Vettore MV (2011). The association between low-socioeconomic status mother’s sense of coherence and their child’s utilization of dental care. Comm Dent Oral Epidemiol..

[29] de Leeuw E, Clavier C, Breton E. Health policy - why research it and how: Health political science. Heal Res Policy Syst. 2014;12(1). Access 7 Dec 2023. 10.1186/1478-4505-12-55 PMC4246431 25248956

[30] Listl S, Baltussen R, Carrasco-Labra A, Carrer FC, Lavis JN (2023). Evidence-informed oral health policy making: opportunities and challenges. J Dent Res.

[31] World Health Organization. Monitoring the Building Blocks of Health Systems : a Handbook of Indicators and. 2010.

[32] McLaren L, Petit R (2018). Universal and targeted policy to achieve health equity: a critical analysis of the example of community water fluoridation cessation in Calgary, Canada in 2011. Crit Public Health.

[33] Shen A, Bernabé E, Sabbah W (2021). Systematic review of intervention studies aiming at reducing inequality in dental caries among children. Int J Environ Res Public Health.

[34] Adams J, White M. Are the stages of change socioeconomically distributed? A scoping review. American Journal of Health Promotion. 2007. 10.4278/0890-1171-21.4.237 17375489

[35] Watt G (2002). The inverse care law today. Lancet.

[36] Yamamoto T, Hanazato M, Hikichi H, Kondo K, Osaka K, Kawachi I, et al. Change in Geographic Accessibility to Dental Clinics Affects Access to Care. J Dent Res. 2023;220345231167771. Available from: http://www.ncbi.nlm.nih.gov/pubmed/37204154. 10.1177/00220345231167771 PMC10286177 37204154

[37] Hanibuchi T, Aida J, Nakade M, Hirai H, Kondo K. Geographical accessibility to dental care in the Japanese elderly. Community Dent Heal. 2011;28(2):128–35. Available from: https://pubmed.ncbi.nlm.nih.gov/21780351/. Cited 2021 Aug 19. 21780351

[38] Curtis B, Evans RW, Sbaraini A, Schwarz E (2007). Geographic location and indirect costs as a barrier to dental treatment: a patient perspective. Aust Dent J.

[39] McLaren L, Patterson SK, Faris P, Chen G, Thawer S, Figueiredo R (2021). Fluoridation cessation and children’s dental caries: a 7-year follow-up evaluation of Grade 2 schoolchildren in Calgary and Edmonton, Canada. Community Dent Oral Epidemiol.

[40] Szklo M, Javier Nieto F. Epidemiology: Beyond the Basics. Jones & Bartlett Learning; 2nd edition; 2006. 489 p.

[41] S. Greenberg R, R. Daniels S, Dana Flanders W, William Eley J, R. Boring J. Medical Epidemiology: Population Health and Effective Health Care. 5th ed. New York: LANGE Basic Science; 2015. 525 p.

[42] Ono S, Sasabuchi Y, Ishimaru M, Ono Y, Matsui H, Yasunaga H (2021). Short-term effects of reduced cost sharing on childhood dental care utilization and dental caries prevention in Japan. Community Dent Oral Epidemiol.

[43] Weiss DJ, Nelson A, Vargas-Ruiz CA, Gligorić K, Bavadekar S, Gabrilovich E (2020). Global maps of travel time to healthcare facilities. Nat Med.

[44] Kelly C, Hulme C, Farragher T, Clarke G (2016). Are differences in travel time or distance to healthcare for adults in global north countries associated with an impact on health outcomes? A systematic review. BMJ Open.

