# Supplementary material for: Associations between school-based fluoride mouth-rinse program, medical-dental expense subsidy policy, and children's oral health in Japan: an ecological study
Source: BMC Public Health. 2024 Mar 12;24:762. doi: 10.1186/s12889-024-18156-y (PMC10929176; doi:10.1186/s12889-024-18156-y)
Supplement: Supplementary file 1 — Supplementary Material 1. [file 12889_2024_18156_MOESM1_ESM.pptx]

## Slide 1
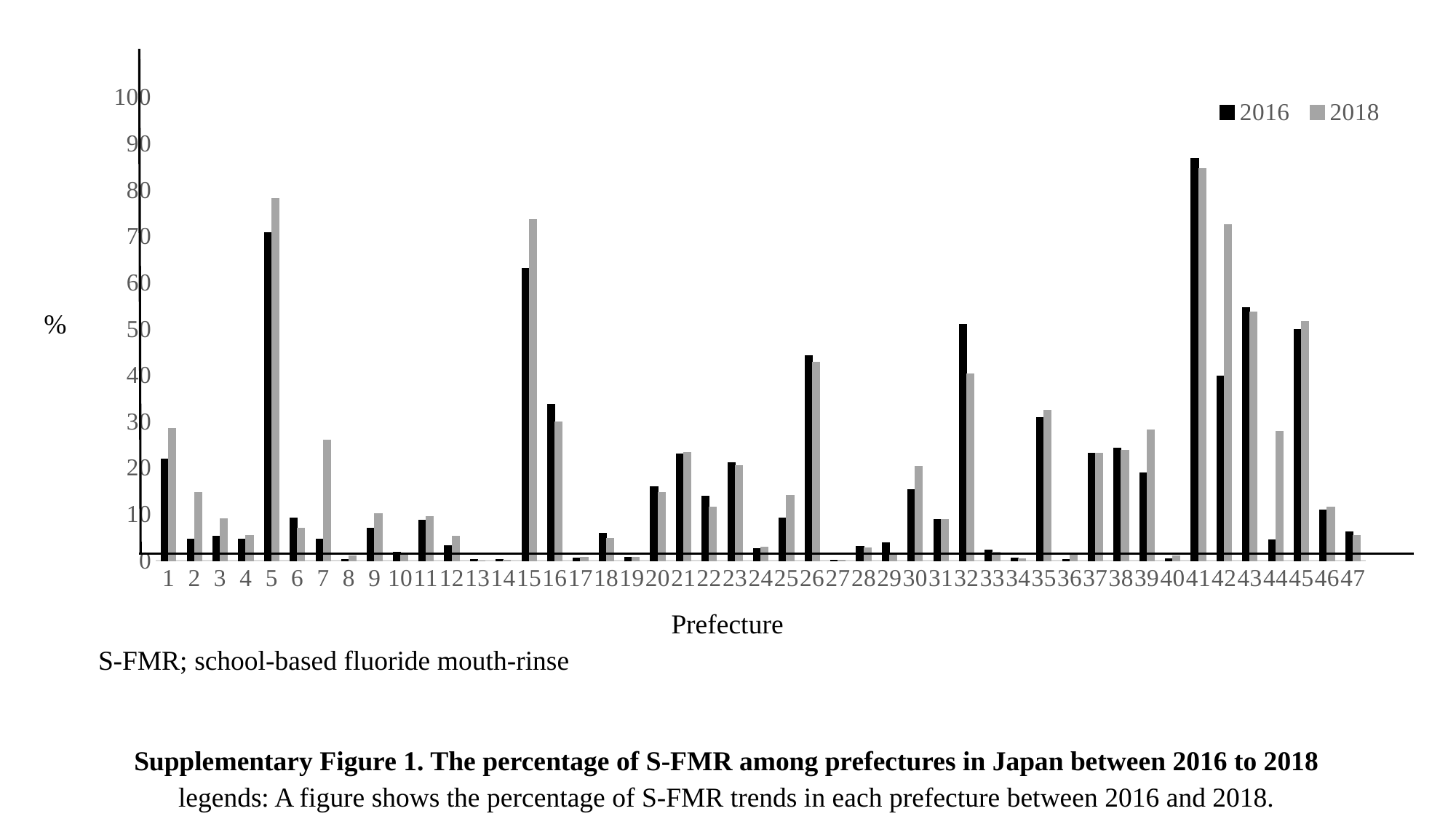

### Chart
| Category | 2016 | 2018 |
|---|---|---|
| 1 | 22.0 | 28.6 |
| 2 | 4.6 | 14.7 |
| 3 | 5.3 | 9.1 |
| 4 | 4.6 | 5.5 |
| 5 | 70.8 | 78.2 |
| 6 | 9.2 | 7.0 |
| 7 | 4.7 | 26.0 |
| 8 | 0.3 | 1.0 |
| 9 | 7.0 | 10.2 |
| 10 | 1.9 | 1.7 |
| 11 | 8.7 | 9.6 |
| 12 | 3.3 | 5.3 |
| 13 | 0.2 | 0.0 |
| 14 | 0.2 | 0.1 |
| 15 | 63.1 | 73.7 |
| 16 | 33.7 | 30.0 |
| 17 | 0.6 | 0.7 |
| 18 | 5.9 | 4.8 |
| 19 | 0.8 | 0.8 |
| 20 | 16.0 | 14.8 |
| 21 | 23.1 | 23.3 |
| 22 | 13.9 | 11.6 |
| 23 | 21.2 | 20.6 |
| 24 | 2.6 | 3.0 |
| 25 | 9.2 | 14.1 |
| 26 | 44.2 | 42.8 |
| 27 | 0.1 | 0.1 |
| 28 | 3.1 | 2.8 |
| 29 | 3.9 | 1.4 |
| 30 | 15.3 | 20.4 |
| 31 | 8.9 | 8.9 |
| 32 | 51.0 | 40.4 |
| 33 | 2.3 | 1.9 |
| 34 | 0.6 | 0.5 |
| 35 | 30.9 | 32.5 |
| 36 | 0.2 | 1.4 |
| 37 | 23.2 | 23.2 |
| 38 | 24.3 | 23.8 |
| 39 | 19.0 | 28.2 |
| 40 | 0.4 | 1.0 |
| 41 | 86.9 | 84.7 |
| 42 | 39.8 | 72.5 |
| 43 | 54.7 | 53.7 |
| 44 | 4.5 | 27.9 |
| 45 | 50.0 | 51.7 |
| 46 | 11.0 | 11.5 |
| 47 | 6.3 | 5.4 |%
Prefecture
S-FMR; school-based fluoride mouth-rinse
Supplementary Figure 1. The percentage of S-FMR among prefectures in Japan between 2016 to 2018
legends: A figure shows the percentage of S-FMR trends in each prefecture between 2016 and 2018.
