# Supplementary material for: Associations between school-based fluoride mouth-rinse program, medical-dental expense subsidy policy, and children's oral health in Japan: an ecological study
Source: BMC Public Health. 2024 Mar 12;24:762. doi: 10.1186/s12889-024-18156-y (PMC10929176; doi:10.1186/s12889-024-18156-y)
Supplement: Supplementary file 2 — Supplementary Material 2. [file 12889_2024_18156_MOESM2_ESM.pptx]

## Slide 1
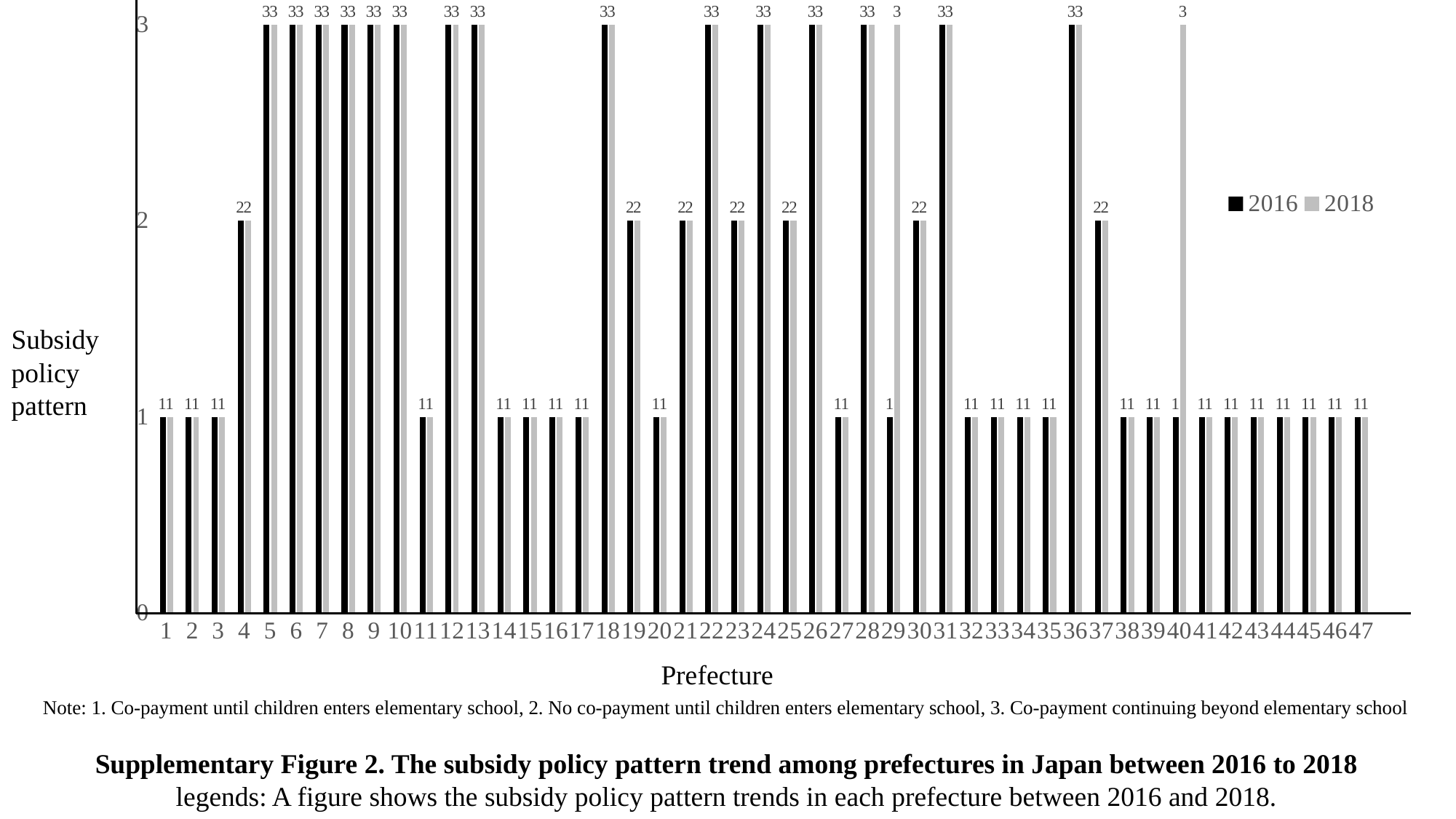

### Chart
| Category | 2016 | 2018 |
|---|---|---|Subsidy
policy
pattern
Prefecture
Note: 1. Co-payment until children enters elementary school, 2. No co-payment until children enters elementary school, 3. Co-payment continuing beyond elementary school
Supplementary Figure 2. The subsidy policy pattern trend among prefectures in Japan between 2016 to 2018
legends: A figure shows the subsidy policy pattern trends in each prefecture between 2016 and 2018.
