# Supplementary material for: Associations between school-based fluoride mouth-rinse program, medical-dental expense subsidy policy, and children's oral health in Japan: an ecological study
Source: BMC Public Health. 2024 Mar 12;24:762. doi: 10.1186/s12889-024-18156-y (PMC10929176; doi:10.1186/s12889-024-18156-y)
Supplement: Supplementary file 3 — Supplementary Material 3. [file 12889_2024_18156_MOESM3_ESM.pptx]

## Slide 1
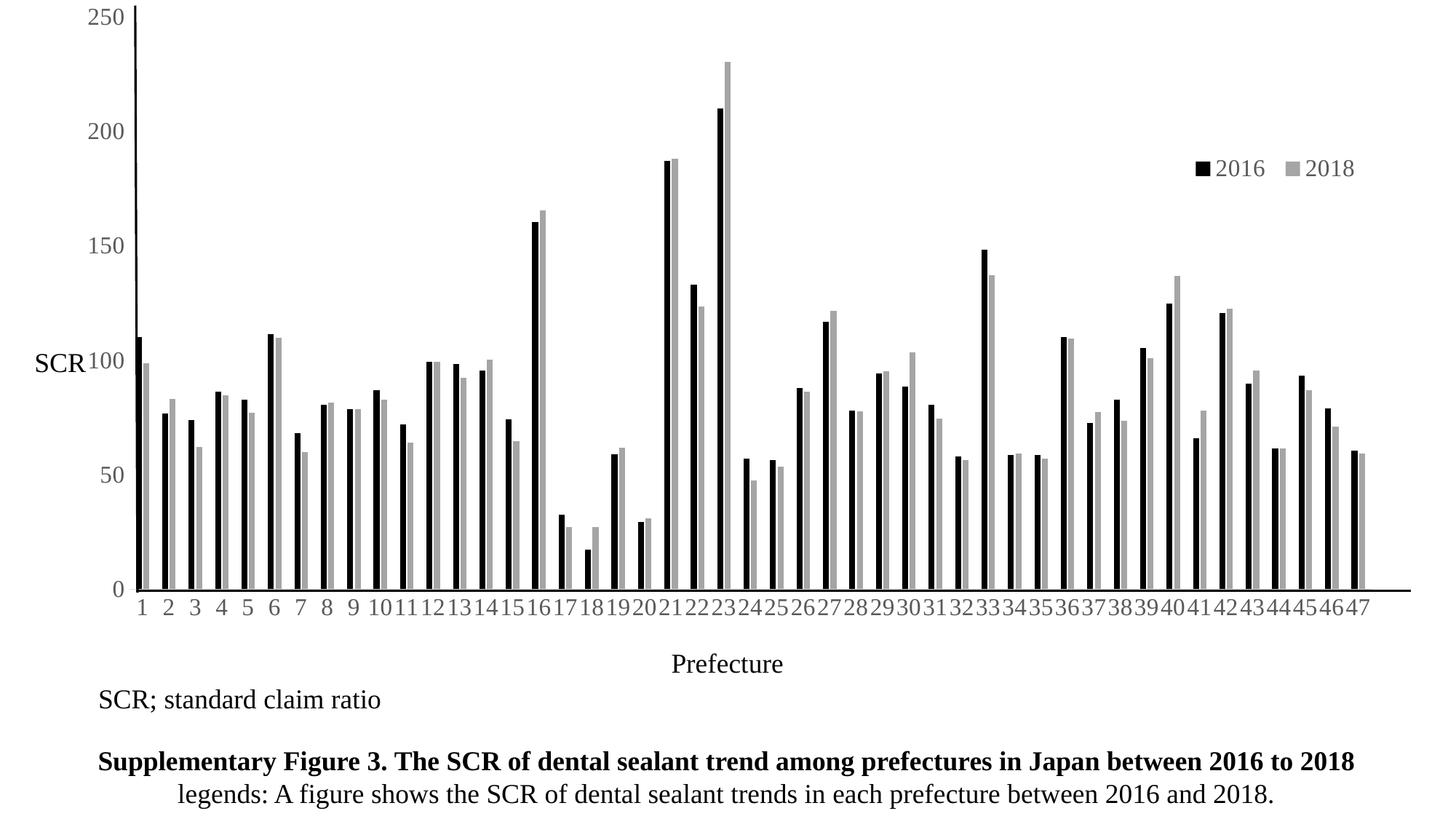

### Chart
| Category | 2016 | 2018 |
|---|---|---|
| 1 | 110.08814801052947 | 98.797212 |
| 2 | 76.92415262526197 | 83.281737 |
| 3 | 73.89843656868477 | 62.091809 |
| 4 | 86.42721471761591 | 84.676645 |
| 5 | 82.77880910527313 | 77.038688 |
| 6 | 111.30084907279172 | 109.79143 |
| 7 | 68.14817990598084 | 59.941688 |
| 8 | 80.58867681132466 | 81.469844 |
| 9 | 78.63027835248924 | 78.596375 |
| 10 | 87.06286957713596 | 82.765281 |
| 11 | 71.93457995355713 | 64.03368 |
| 12 | 99.36302613431843 | 99.187318 |
| 13 | 98.52927164321778 | 92.293881 |
| 14 | 95.38795037857649 | 100.35664 |
| 15 | 74.22974913792821 | 64.544198 |
| 16 | 160.28214951441913 | 165.42227 |
| 17 | 32.48495644692633 | 27.221311 |
| 18 | 17.392371016363864 | 27.117438 |
| 19 | 58.95074649516761 | 61.900043 |
| 20 | 29.53841027625558 | 30.916022 |
| 21 | 187.12589580531346 | 187.96236 |
| 22 | 133.00863521009944 | 123.52977 |
| 23 | 210.03710085676198 | 230.36708 |
| 24 | 57.05456689472441 | 47.365287 |
| 25 | 56.405455629193376 | 53.590699 |
| 26 | 87.96423819450978 | 86.227984 |
| 27 | 116.68366698226464 | 121.69179 |
| 28 | 78.07588710938643 | 77.73753 |
| 29 | 94.3884409502236 | 95.256993 |
| 30 | 88.40311321544331 | 103.39029 |
| 31 | 80.46171640378566 | 74.541892 |
| 32 | 57.96711192263838 | 56.413006 |
| 33 | 148.30490310206488 | 137.14388 |
| 34 | 58.53800484048325 | 59.371248 |
| 35 | 58.720110309431085 | 56.969222 |
| 36 | 110.28707469205821 | 109.5925 |
| 37 | 72.71915314247687 | 77.307887 |
| 38 | 82.90973982965637 | 73.486678 |
| 39 | 105.44594369932203 | 101.08328 |
| 40 | 124.92213310697646 | 137.00036 |
| 41 | 65.99196844839427 | 78.2066 |
| 42 | 120.55428678635298 | 122.57802 |
| 43 | 89.8432623057022 | 95.622503 |
| 44 | 61.62083337845865 | 61.434447 |
| 45 | 93.33134862849968 | 86.8307 |
| 46 | 78.89839059613611 | 71.051421 |
| 47 | 60.45150473903088 | 59.362151 |SCR
Prefecture
SCR; standard claim ratio
Supplementary Figure 3. The SCR of dental sealant trend among prefectures in Japan between 2016 to 2018
legends: A figure shows the SCR of dental sealant trends in each prefecture between 2016 and 2018.
