# Supplementary material for: Associations between school-based fluoride mouth-rinse program, medical-dental expense subsidy policy, and children's oral health in Japan: an ecological study
Source: BMC Public Health. 2024 Mar 12;24:762. doi: 10.1186/s12889-024-18156-y (PMC10929176; doi:10.1186/s12889-024-18156-y)
Supplement: Supplementary file 4 — Supplementary Material 4. [file 12889_2024_18156_MOESM4_ESM.pptx]

## Slide 1
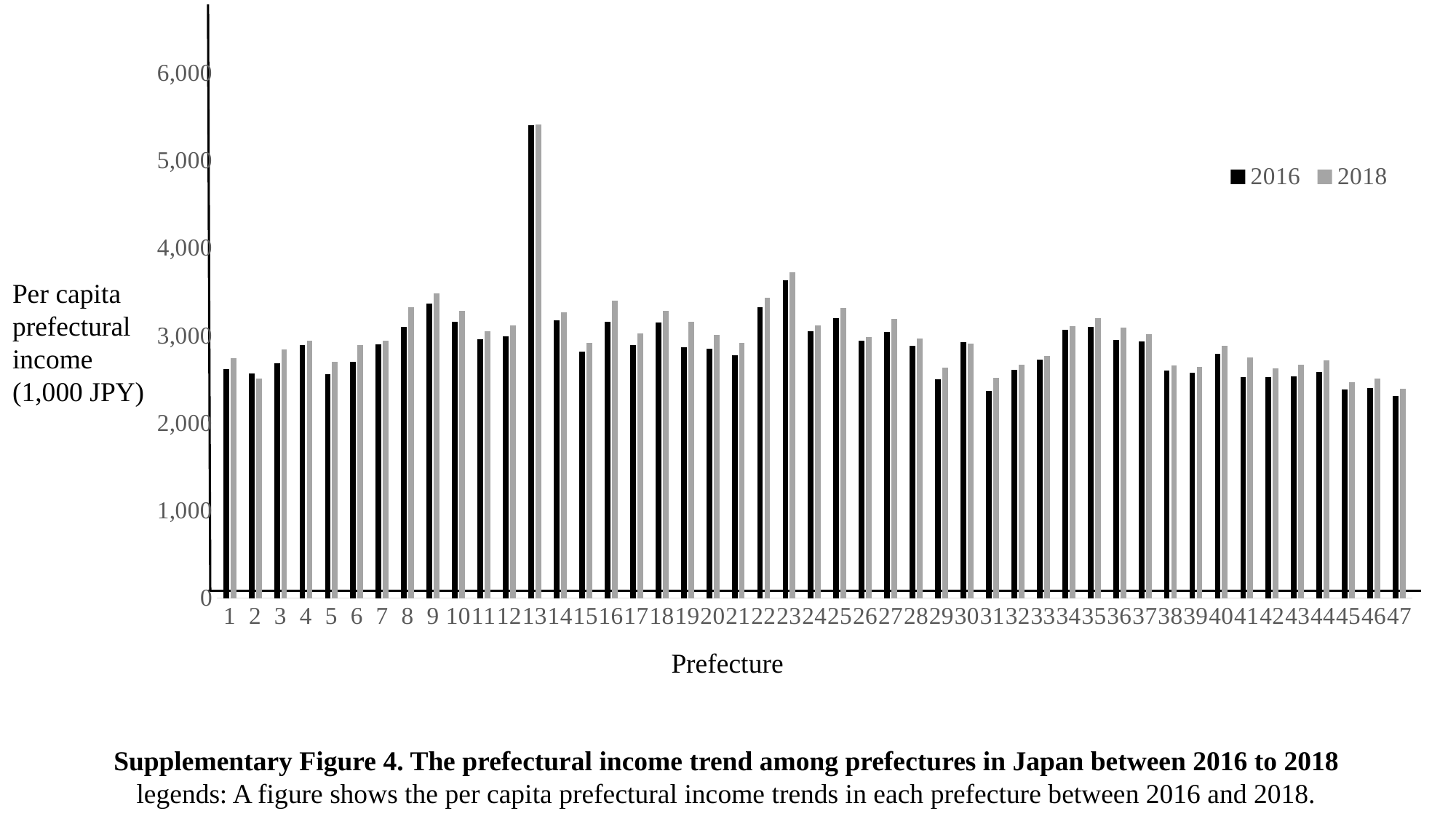

### Chart
| Category | 2016 | 2018 |
|---|---|---|
| 1 | 2615.391413924364 | 2742.2189 |
| 2 | 2570.3224659249922 | 2507.4137 |
| 3 | 2683.8192324405577 | 2841.0701 |
| 4 | 2890.464868762124 | 2944.8764 |
| 5 | 2559.557974502033 | 2697.1609 |
| 6 | 2698.7024631010977 | 2896.5629 |
| 7 | 2902.415875754961 | 2942.7718 |
| 8 | 3099.460853339025 | 3327.3051 |
| 9 | 3369.38717172457 | 3479.1938 |
| 10 | 3159.5741760755395 | 3282.8785 |
| 11 | 2960.7471312224866 | 3046.8394 |
| 12 | 2993.087732380758 | 3115.8336 |
| 13 | 5400.274017708684 | 5414.8311 |
| 14 | 3173.3501346819908 | 3267.8298 |
| 15 | 2817.4783469535687 | 2915.9098 |
| 16 | 3160.738094722093 | 3398.1409 |
| 17 | 2890.1430038631374 | 3022.6947 |
| 18 | 3149.9199269948917 | 3279.803 |
| 19 | 2865.657556634382 | 3159.7409 |
| 20 | 2848.299262714523 | 3009.7635 |
| 21 | 2774.2043017559963 | 2919.0055 |
| 22 | 3321.941400364675 | 3431.6253 |
| 23 | 3629.532430164249 | 3727.813 |
| 24 | 3046.196403566791 | 3120.941 |
| 25 | 3200.6844418649093 | 3318.4682 |
| 26 | 2938.689979730163 | 2982.6712 |
| 27 | 3041.1828480957624 | 3189.5069 |
| 28 | 2882.814250747695 | 2967.7493 |
| 29 | 2501.5523634189303 | 2631.5064 |
| 30 | 2924.631006076437 | 2912.7448 |
| 31 | 2370.9305877932557 | 2515.3311 |
| 32 | 2606.1384855561205 | 2667.2549 |
| 33 | 2723.2297634461615 | 2769.0599 |
| 34 | 3065.6556756520527 | 3109.1331 |
| 35 | 3096.046328169822 | 3198.54 |
| 36 | 2950.840869342661 | 3092.3529 |
| 37 | 2935.8263905533618 | 3013.4912 |
| 38 | 2598.3508786731386 | 2658.0154 |
| 39 | 2573.1456977524786 | 2643.8157 |
| 40 | 2793.0822428914184 | 2885.2805 |
| 41 | 2528.8730022489976 | 2752.597 |
| 42 | 2522.780349899619 | 2629.178 |
| 43 | 2533.2911729876187 | 2667.4459 |
| 44 | 2585.420365409173 | 2713.6654 |
| 45 | 2387.6010221033034 | 2467.5702 |
| 46 | 2398.182809865061 | 2508.548 |
| 47 | 2313.106441989303 | 2390.9687 |Per capita prefectural income (1,000 JPY)
Prefecture
Supplementary Figure 4. The prefectural income trend among prefectures in Japan between 2016 to 2018
legends: A figure shows the per capita prefectural income trends in each prefecture between 2016 and 2018.
