# Supplementary material for: Associations between school-based fluoride mouth-rinse program, medical-dental expense subsidy policy, and children's oral health in Japan: an ecological study
Source: BMC Public Health. 2024 Mar 12;24:762. doi: 10.1186/s12889-024-18156-y (PMC10929176; doi:10.1186/s12889-024-18156-y)
Supplement: Supplementary file 5 — Supplementary Material 5. [file 12889_2024_18156_MOESM5_ESM.pptx]

## Slide 1
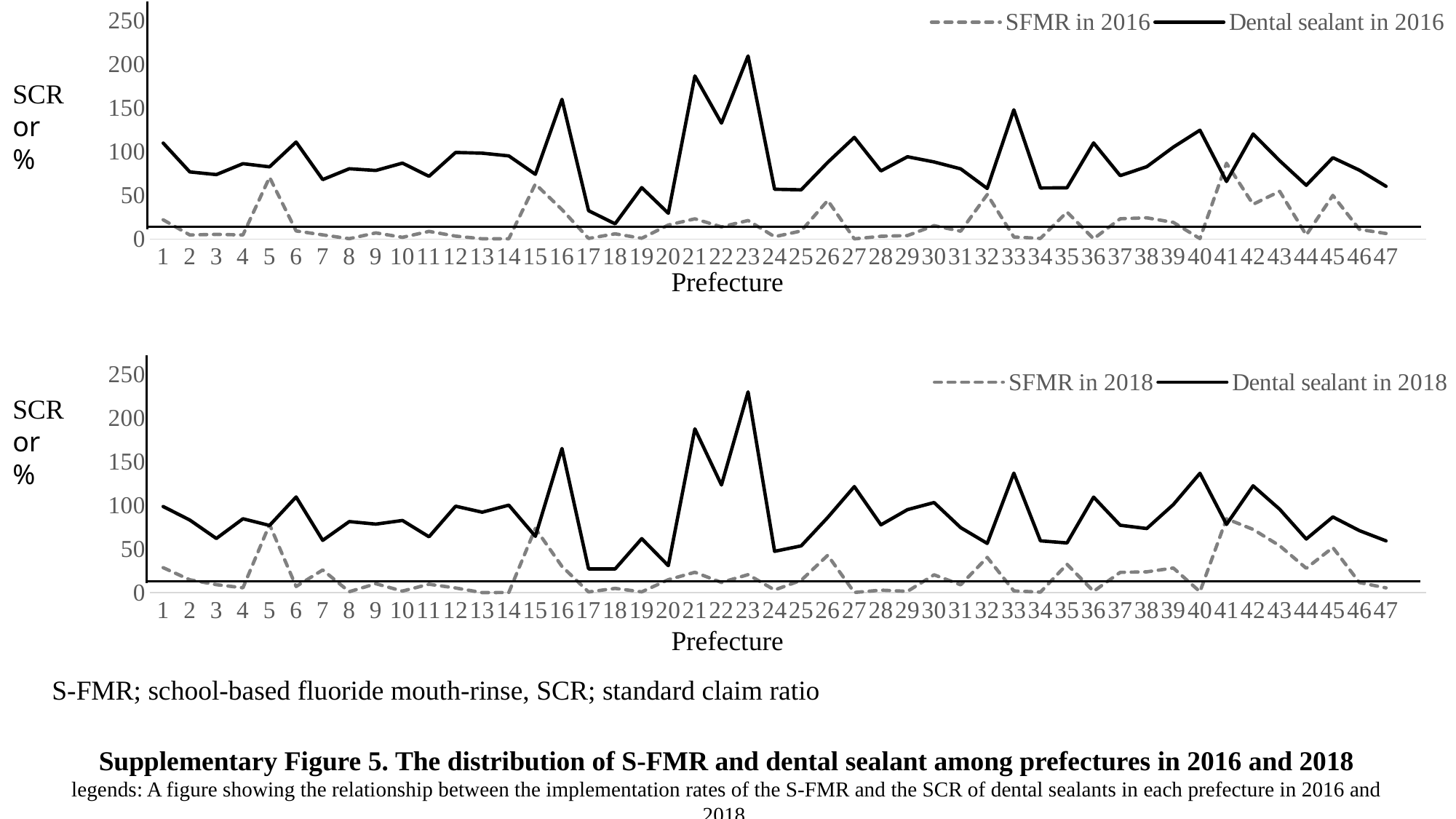

### Chart
| Category | SFMR in 2016 | Dental sealant in 2016 |
|---|---|---|
| 1 | 22.0 | 110.08814801052947 |
| 2 | 4.6 | 76.92415262526197 |
| 3 | 5.3 | 73.89843656868477 |
| 4 | 4.6 | 86.42721471761591 |
| 5 | 70.8 | 82.77880910527313 |
| 6 | 9.2 | 111.30084907279172 |
| 7 | 4.7 | 68.14817990598084 |
| 8 | 0.3 | 80.58867681132466 |
| 9 | 7.0 | 78.63027835248924 |
| 10 | 1.9 | 87.06286957713596 |
| 11 | 8.7 | 71.93457995355713 |
| 12 | 3.3 | 99.36302613431843 |
| 13 | 0.2 | 98.52927164321778 |
| 14 | 0.2 | 95.38795037857649 |
| 15 | 63.1 | 74.22974913792821 |
| 16 | 33.7 | 160.28214951441913 |
| 17 | 0.6 | 32.48495644692633 |
| 18 | 5.9 | 17.392371016363864 |
| 19 | 0.8 | 58.95074649516761 |
| 20 | 16.0 | 29.53841027625558 |
| 21 | 23.1 | 187.12589580531346 |
| 22 | 13.9 | 133.00863521009944 |
| 23 | 21.2 | 210.03710085676198 |
| 24 | 2.6 | 57.05456689472441 |
| 25 | 9.2 | 56.405455629193376 |
| 26 | 44.2 | 87.96423819450978 |
| 27 | 0.1 | 116.68366698226464 |
| 28 | 3.1 | 78.07588710938643 |
| 29 | 3.9 | 94.3884409502236 |
| 30 | 15.3 | 88.40311321544331 |
| 31 | 8.9 | 80.46171640378566 |
| 32 | 51.0 | 57.96711192263838 |
| 33 | 2.3 | 148.30490310206488 |
| 34 | 0.6 | 58.53800484048325 |
| 35 | 30.9 | 58.720110309431085 |
| 36 | 0.2 | 110.28707469205821 |
| 37 | 23.2 | 72.71915314247687 |
| 38 | 24.3 | 82.90973982965637 |
| 39 | 19.0 | 105.44594369932203 |
| 40 | 0.4 | 124.92213310697646 |
| 41 | 86.9 | 65.99196844839427 |
| 42 | 39.8 | 120.55428678635298 |
| 43 | 54.7 | 89.8432623057022 |
| 44 | 4.5 | 61.62083337845865 |
| 45 | 50.0 | 93.33134862849968 |
| 46 | 11.0 | 78.89839059613611 |
| 47 | 6.3 | 60.45150473903088 |
| | None | None |SCR
or
%
Prefecture
### Chart
| Category | SFMR in 2018 | Dental sealant in 2018 |
|---|---|---|
| 1 | 28.6 | 98.797212 |
| 2 | 14.7 | 83.281737 |
| 3 | 9.1 | 62.091809 |
| 4 | 5.5 | 84.676645 |
| 5 | 78.2 | 77.038688 |
| 6 | 7.0 | 109.79143 |
| 7 | 26.0 | 59.941688 |
| 8 | 1.0 | 81.469844 |
| 9 | 10.2 | 78.596375 |
| 10 | 1.7 | 82.765281 |
| 11 | 9.6 | 64.03368 |
| 12 | 5.3 | 99.187318 |
| 13 | 0.0 | 92.293881 |
| 14 | 0.1 | 100.35664 |
| 15 | 73.7 | 64.544198 |
| 16 | 30.0 | 165.42227 |
| 17 | 0.7 | 27.221311 |
| 18 | 4.8 | 27.117438 |
| 19 | 0.8 | 61.900043 |
| 20 | 14.8 | 30.916022 |
| 21 | 23.3 | 187.96236 |
| 22 | 11.6 | 123.52977 |
| 23 | 20.6 | 230.36708 |
| 24 | 3.0 | 47.365287 |
| 25 | 14.1 | 53.590699 |
| 26 | 42.8 | 86.227984 |
| 27 | 0.1 | 121.69179 |
| 28 | 2.8 | 77.73753 |
| 29 | 1.4 | 95.256993 |
| 30 | 20.4 | 103.39029 |
| 31 | 8.9 | 74.541892 |
| 32 | 40.4 | 56.413006 |
| 33 | 1.9 | 137.14388 |
| 34 | 0.5 | 59.371248 |
| 35 | 32.5 | 56.969222 |
| 36 | 1.4 | 109.5925 |
| 37 | 23.2 | 77.307887 |
| 38 | 23.8 | 73.486678 |
| 39 | 28.2 | 101.08328 |
| 40 | 1.0 | 137.00036 |
| 41 | 84.7 | 78.2066 |
| 42 | 72.5 | 122.57802 |
| 43 | 53.7 | 95.622503 |
| 44 | 27.9 | 61.434447 |
| 45 | 51.7 | 86.8307 |
| 46 | 11.5 | 71.051421 |
| 47 | 5.4 | 59.362151 |
| | None | None |SCR
or
%
Prefecture
S-FMR; school-based fluoride mouth-rinse, SCR; standard claim ratio
Supplementary Figure 5. The distribution of S-FMR and dental sealant among prefectures in 2016 and 2018
legends: A figure showing the relationship between the implementation rates of the S-FMR and the SCR of dental sealants in each prefecture in 2016 and 2018.
