# Supplementary material for: Associations between school-based fluoride mouth-rinse program, medical-dental expense subsidy policy, and children's oral health in Japan: an ecological study
Source: BMC Public Health. 2024 Mar 12;24:762. doi: 10.1186/s12889-024-18156-y (PMC10929176; doi:10.1186/s12889-024-18156-y)
Supplement: Supplementary file 6 — Supplementary Material 6. [file 12889_2024_18156_MOESM6_ESM.docx]

**Tables**

Supplementary Table 1. The associations between S-FMR and medical-dental expense subsidy policy on the SCR of deciduous tooth extraction adjusting for the public assistance rate.

Supplementary Table 2. The associations between S-FMR and medical-dental expense subsidy policy on children's D (decayed teeth) instead of DMFT.

Supplementary Table 1. The associations between S-FMR and medical-dental expense subsidy policy on the SCR of deciduous tooth extraction adjusting for the public assistance rate.

|  |  | Coef. † | 95%CI |
| --- | --- | --- | --- |
| The rate of S-FMR utilization (per person) (%) | | -0.11 | (-0.20; -0.01)* |
| Subsidy policy | No co-payment until children enters elementary school | 11.42 | (3.30;19.54)* |
| (REF: Co-pay until children enters elementary school) | Co-payment continuing beyond elementary school | 2.19 | (-1.67;6.04) |
| SCR of dental sealants | | 0.12 | (0.06;0.19) |
| Per capita prefectural income (1,000 JPY) | | 0.01 | (0.0007;0.020)* |
| The rate of Industrial Structure 3 (Service / Commerce) (%) | | 0.50 | (0.03;0.98)* |
| Number of families (per household) | | 15.51 | (-2.59;33.6) |
| Percentage of the nuclear family (%) | | -0.24 | (-1.06;0.58) |
| Percentage of households living with older adults (%) | | 0.40 | (-0.25;1.04) |
| Total fertility rate (%) | | -11.57 | (-31.69;8.55) |
| Percentage of college graduates (%) | | -0.78 | (-1.70;0.14) |
| Percentage of receiving public assistance (%) | | 0.29 | (-1.62;2.19) |
| The unemployment rate (%) | | 1.07 | (-0.30;2.44) |
| The number of dentists working in private dental office (per 100,000 people) | | 0.08 | (-0.14;0.30) |

†: Coefficient was estimated using a linear regression model with all variables simultaneously entered into the model.

*: It means P<0.05

Abbreviations: Coef.; Coefficient, S-FMR; school-based fluoride mouth-rinse, SCR; standardized claim ratio, CI; confidence interval, REF; reference

legends: The impact of S-FMR and medical-dental expense subsidy policy on the SCR of deciduous tooth extraction was analyzed, adding the public assistance rate into the model as a proxy indicator of SES at the regional level.

Supplementary Table 2. The associations between S-FMR and medical-dental expense subsidy policy on children's D (decayed teeth) instead of DMFT.

|  |  | Coef. † | 95%CI |
| --- | --- | --- | --- |
| The rate of S-FMR utilization (per person) (%) | | -0.002 | (-0.003; -0.001)* |
| Subsidy policy | No co-payment until children enters elementary school | 0.03 | (-0.03;0.08) |
| (REF: Co-pay until children enters elementary school) | Co-payment continuing beyond elementary school | 0.01 | (-0.04;0.05) |
| SCR of dental sealants | | -0.0005 | (-0.001;0.00002) |
| Per capita prefectural income (1,000 JPY) | | 0.0001 | (-0.00001;0.0001) |
| The rate of Industrial Structure 3 (Service / Commerce) (%) | | 0.010 | (0.004;0.017)* |
| Number of families (per household) | | 0.04 | (-0.13;0.20) |
| Percentage of the nuclear family (%) | | 0.005 | (-0.002;0.013) |
| Percentage of households living with older adults (%) | | -0.005 | (-0.012;0.002) |
| Total fertility rate (%) | | 0.15 | (-0.03;0.33) |
| Percentage of college graduates (%) | | -0.02 | (-0.03; -0.01)* |
| Percentage of receiving public assistance (%) | | 0.04 | (0.001;0.076)* |
| The unemployment rate (%) | | 0.04 | (0.02;0.06)* |
| The number of dentists working in private dental office (per 100,000 people) | | -0.001 | (-0.003;0.001) |

†: Coefficient was estimated using a linear regression model with all variables simultaneously entered into the model.

*: It means P<0.05

Abbreviations: DMFT; decayed, missing, or filled permanent teeth, Coef.; Coefficient, S-FMR; school-based fluoride mouth-rinse, SCR; standardized claim ratio, CI; confidence interval, REF; reference

legends: The analysis was conducted to identify predictors for D (decayed teeth) and to examine the presence of interaction terms.
